# Epidemiologic and Genomic Characterizations of Porcine Kobuviruses in Diarrheic and Healthy Pigs

**DOI:** 10.3390/ani13193129

**Published:** 2023-10-07

**Authors:** Yu Zang, Binghui Feng, Zitao Huang, Dashi Zhao, Wenhao Qi, Yuejia Qiu, Ming Qiu, Chen Li, Hong Lin, Wanglong Zheng, Jianzhong Zhu, Nanhua Chen

**Affiliations:** 1 College of Veterinary Medicine, Yangzhou University, Yangzhou 225009, China; zangyu20010522@163.com (Y.Z.); fengbinghui0421@163.com (B.F.); 18246986065@163.com (D.Z.); qiwenhao2829@163.com (W.Q.); qiuyuejia2022@163.com (Y.Q.); qiuming1997@126.com (M.Q.); lichen19991104@163.com (C.L.); linhonglynn@yzu.edu.cn (H.L.); wanglongzheng@yzu.edu.cn (W.Z.); jzzhu@yzu.edu.cn (J.Z.); 2 Animal Health Supervision Institute of Fengxi District, Chaozhou 521031, China; 13450818935@163.com; 3 Joint International Research Laboratory of Agriculture and Agri-Product Safety, Yangzhou 225009, China; 4 International Research Laboratory of Prevention and Control of Important Animal Infectious Diseases and Zoonotic Diseases of Jiangsu Higher Education Institutions, Yangzhou 225009, China; 5 Comparative Medicine Research Institute, Yangzhou University, Yangzhou 225009, China; 6 Jiangsu Co-Innovation Center for Prevention and Control of Important Animal Infectious Diseases and Zoonoses, Yangzhou University, Yangzhou 225009, China; 7 Key Laboratory of Animal Pathogen Infection and Immunology of Fujian Province, Fuzhou 350002, China; 8 Fujian Provincial Key Laboratory for Prevention and Control of Animal Infectious Diseases and Biotechnology, Longyan University, Longyan 364012, China

**Keywords:** porcine kobuvirus, epidemiology, evolution, polyprotein, diarrheic and healthy pigs

## Abstract

**Simple Summary:**

Enteric diseases pose huge threats to the global swine industry. Porcine kobuvirus (PKV) is a newly emerging enteric virus, but the role of PKV in causing enteric diseases is unclear. Here, we evaluated the up-to-date prevalence and evolution of PKV in both diarrheic and healthy pigs. Our results showed that PKV was highly prevalent in Chinese swine herds during 2018–2022. In addition, PKV is frequently co-infected with porcine epidemic diarrhea virus (PEDV). Most PKV and PEDV double-positive pigs were clinically diseased, while most PKV-positive but PEDV-negative pigs were clinically healthy, suggesting that PKV is unlikely to be a direct gastroenteritis-causing virus but a potential opportunistic pathogen. Furthermore, three PKV genomes were obtained and submitted for evolutionary evaluation. The results indicated that Chinese PKV has evolved into three groups, and SH-W-CHN-like viruses are predominant. Moreover, even though mutations frequently occurred during the evolution of PKV in China, large amounts of them are nonsense and strong negative selection seems to play a critical role in maintaining the antigenic stability. Overall, this study not only provides an epidemiological clue on the role of PKV in enteric diseases, but also the evolutionary status of PKV in both diarrheic and healthy pigs in China.

**Abstract:**

Porcine kobuvirus (PKV) is an enteric virus commonly detected in both diarrheic and healthy pigs. Little is known about the role of PKV in enteric diseases. In this study, an epidemiological investigation based on 324 intestinal samples collected from six provinces of China during the period of 2018 to 2022 was performed, and showed that PKV has an overall 65.43% (212/324) positive rate. Noticeably, 89.47% (17/19) of PKV and porcine epidemic diarrhea virus (PEDV) double-positive pigs were clinically diseased, while 91.71% (177/193) of PKV-positive but PEDV-negative pigs were clinically healthy, suggesting that PKV infection in itself is unlikely to cause enteric diseases. In addition, three PKV genomes were obtained from both diseased and healthy pigs. Phylogenetic analysis showed that Chinese PKV strains could be divided into three groups (SH-W-CHN-like, S-1-HUN-like and JXAT2015-like strains). All three obtained PKV genomes belong to SH-W-CHN-like strains and JSYZ1806-158 was detected as a recombinant virus. Furthermore, multiple comparisons showed that nucleotide similarities are clearly lower than amino acid similarities for PKV polyproteins. Selective pressure analysis indicated that Chinese PKV polyproteins are predominantly under negative selection. Overall, this study provided new insights into the prevalence and evolution of PKV in both diarrheic and healthy pigs in China.

## 1. Introduction

Kobuviruses are positive-sense single-stranded RNA viruses belonging to the genus of *Kobuvirus* in the family of *Piconaviridae* [1]. The genomes of kobuviruses are ~8.2–8.4 kb and contain a 5′ untranslated region (UTR), a single open reading frame (ORF), a 3′ UTR and a polyA tail [2]. The ORF encodes a polyprotein consisting of a leader protein (L), three structural capsid proteins (VP0, VP3 and VP1) and seven non-structural proteins (2A, 2B, 2C, 3A, 3B, 3C and 3D) [3,4]. Kobuviruses can be divided into three species: *Aichivirus A*, *Aichivirus B* and *Aichivirus C* [5]. The prototype isolates of these three species are human Aichivirus A846/88 strain [6], bovine kobuvirus U-1 strain [7] and porcine kobuvirus (PKV) S-1-HUN strain [1], respectively.

Kobuviruses have a wide range of hosts, including human, cattle, pig, sheep, goat, dog, cat, rabbit, bird, ferret, rodent and bat [8,9,10]. An epidemiological investigation in Vietnam indicated that frequent cross-species transmission of Kobuviruses could be detected both within and among mammalian species [11]. But PKV is only found in pigs [12]. PKV was first discovered in Hungary in 2007 [1] and subsequently found in countries all around the world, including China [13], Thailand [14], Japan [15], South Korea [16], Brazil and The Netherlands [17], Italy [18], the United States [19], the Czech Republic [20], Kenya [21], Vietnam [22], Slovakia [23], Ireland [24], Denmark [25], Belgium [26], Hungary [27], Serbia [8], Mexico [28] and India [29]. Besides the wide spread of PKV all around the world, its prevalence rate is also high, ranging from 13% to 99% in domestic pigs [8,9,14,21].

However, the pathogenic capability of PKV in pigs is still questioned. A statistical analysis showed that 84.5% (71/84) of samples from diarrheic pigs were PKV-positive, but only 19.3% (16/83) samples from control pigs were PKV-positive, suggesting that PKV infection is closely correlated with gastrointestinal diseases in pigs [30]. In contrast, another study showed that samples from control pigs (33.8%, 49/145) have an even higher PKV-positive rate than samples from diarrheic pigs (24.7%, 59/239) [27]. Therefore, whether PKV is a directly causative virus for gastrointestinal diseases in young pigs deserves further investigation.

Several swine enteric viruses, such as porcine epidemic diarrhea virus (PEDV) and transmissible gastroenteritis virus (TGEV), have been confirmed as direct causative pathogens for diarrhea in neonatal piglets [31]. PKV is also an enteric virus, but its exact role in diarrheic diseases is still unclear. Our previous metagenomic and pathogenic analyses showed that PKV is the most abundant virus (58.33%), followed by PEDV (34.45%) in several diarrheic neonatal piglets [32]. Furthermore, significant in vivo replications of PKV and PEDV are concurrent with diarrheic diseases in neonatal piglets. Organ distribution analysis also showed that intestinal tissues have the highest virus loads of PKV and PEDV, suggesting that PKV and PEDV are closely associated with neonatal diarrheic diseases.

To determine the prevalence of PKV in China, here, we performed an epidemiological investigation based on 324 intestinal samples (64 from diarrheic piglets < 10 days old and 260 from pigs > one month old without enteric diseases) collected from six provinces of China in 2018–2022. The correlations between PKV infection, PEDV infection and clinical diseases were also evaluated. To evaluate the evolution of PKV in Chinese swine herds, three nearly complete genomes were obtained from diarrheic and healthy pigs and submitted for phylogenetic analysis, multiple comparison, recombination detection and selective pressure analysis.

## 2. Materials and Methods

### 2.1. Clinical Sample Collection

A total of 324 intestinal samples (duodenum or jejunum based on the availability of clinical samples) were submitted to our laboratory from six provinces of China (Shandong, Henan, Jiangsu, Fujian, Guangdong and Xinjiang) from April 2018 to February 2022. Out of 324 samples, 64 were collected from clinically diseased piglets up to the age of 10 days old. Their symptoms mainly included diarrhea, anorexia, vomiting and death. The other 260 samples were collected from pigs (>one month old) without enteric diseases. This study did not deal with live animals and did not require animal ethics approval.

### 2.2. Real-Time PCR Detection and Genomic Sequencing

**Figure 1:**
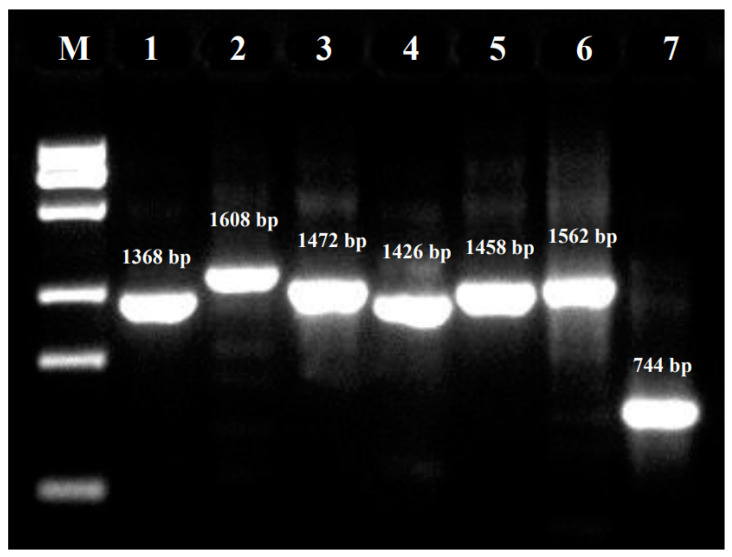
PKV genome amplification. The PCR amplicons of GDCZ2202-1606 sample covering the PKV genome using primers in Table 1 are presented. The PKV genome was divided into seven overlapping fragments. The size of each amplicon is shown.

To analyze the infection rate of PKV and PEDV in Chinese swine herds in recent years, total RNAs were extracted from these clinically intestinal samples using TRIpure Reagent (Aidlab, Beijing, China) according to the manufacturer’s instructions. cDNAs were produced via reverse transcription using HiScript III 1st Strand cDNA Synthesis Kit (+gDNA wiper, Vazyme, Nanjing, China). The obtained sample cDNAs were tested using our previously described PKV and PEDV real-time RT-PCR (qPCR) assays [32] using StepOne Plus Real-time PCR System (Thermo Fisher Scientific, Waltham, MA, USA) and Q711-00 hamQ Universal SYBR qPCR Master Mix (Vazyme, China). In addition, all PKV qPCR amplicons were further confirmed through Sanger sequencing (Genewiz, Suzhou, China). Three PKV-positive samples from three cities of three provinces (Yangzhou city of Jiangsu province, Kashi city of Xinjiang province, Chaozhou city of Guangdong province) and from the period of 2018 to 2022 were selected as representative samples and submitted for genome sequencing (Table 1). The PKV genome amplification of a representative sample (GDCZ2202-1606) containing seven overlapping fragments is shown in Figure 1.

### 2.3. Sequence Alignment and Phylogenetic Analysis

To evaluate the up-to-date evolution of Chinese PKV, multiple sequence alignment was performed using ClustalX 2.0 (Conway Institute, University College Dublin, UK) based on three PKV polyprotein sequences obtained in this study, thirty available Chinese PKV polyprotein sequences, and three representative polyprotein sequences from Archiviruses A, B and C. The phylogenetic tree was constructed based on the aligned sequences using MEGA 6.06 (Pennsylvania State University, State College, PA, USA) with neighbor-joining method and maximum composite likelihood model, which included transitions and transversions, substitutions, homogeneous patterns among lineages and uniform rates among sites [33]. The robustness of the phylogenetic tree was evaluated through bootstrapping with 1000 replicates.

### 2.4. Recombination Detection and Selective Pressure Analysis

Recombination events within PKV polyprotein-encoding sequences were detected using RDP4, as we have previously reported [34]. Briefly, seven methods embedded in RDP version 4 software, RDP, GENECONV, BootScan, MaxChi, Chimaera, SiScan and 3Seq, were utilized to detect recombination events and breakpoints. The default settings were used for all seven methods. The highest acceptable *p*-value was set at 0.05, as described previously [35]. The potential recombination events detected using RDP4 were further evaluated using SimPlot 3.5.1 (Johns Hopkins University, Baltimore, MD, USA).

Selective pressure analysis was performed based on all available Chinese PKV polyprotein-encoding sequences. The selective pressure was evaluated through the ratio of dN/dS, as previously described [36]. When the dN/dS ratio was higher, equal to or lower than 1, it was considered positive, neutral, and negative selection, respectively. The alignment was evaluated for selection using the single-likelihood ancestor counting (SLAC), fixed effects likelihood (FEL) and a fast, unconstrained Bayesian approximation for inferring selection (FUBAR) methods, provided by the Data-Monkey Web Server (http://www.datamonkey.org, accessed on 15 August 2023) [37]. Sites were considered under selective pressure only when detected by at least two methods. The significance level was set at *p* < 0.05 for SLAC and FEL and a posterior probability higher than 0.95 for FUBAR as previously described [38].

## 3. Results

### 3.1. PKV Is Highly Prevalent in Chinese Swine Herds

**Figure 2:**
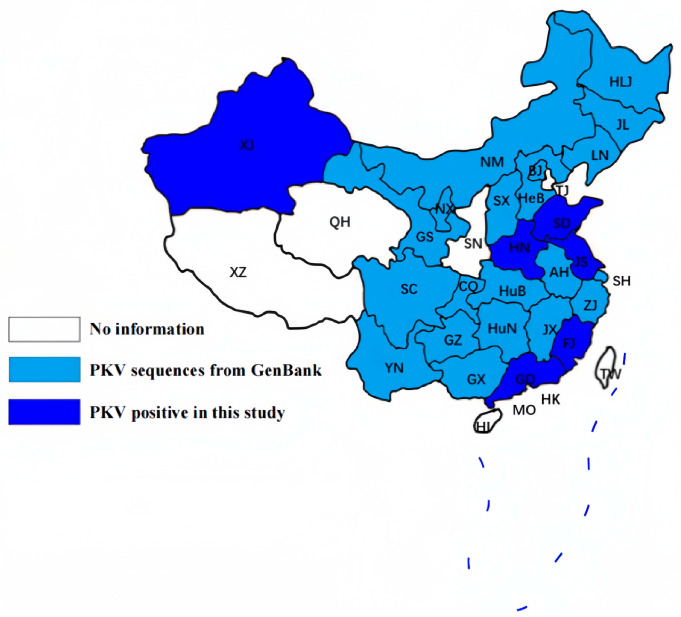
Geographical distribution of PKV in China. Retrospective evaluation indicated that PKV has been detected in at least 26 provinces/cities of China. The six provinces with PKV-positive samples detected in this study are highlighted in dark blue, while the other provinces/cities with PKV sequences from GenBank are marked in light blue.

Real-time RT-PCR results showed that the overall PKV-positive rate is 65.43% (212/324) (Table 2). In addition, PKV could be detected in all six provinces, with positive rates from 21.43% to 82.14%. Meanwhile, PKV could be detected every year from 2018 to 2022, with positive rates of 25.00–87.29%. Furthermore, retrospective analysis based on our results and Chinese PKV sequences available from GenBank suggested that PKV has been detected in at least 26 provinces/cities of China (Figure 2).

### 3.2. PKV Is Unlikely to Be a Direct Causative Pathogen for Enteric Diseases

To evaluate the correlations of PKV infection, PEDV infection and clinical diseases, we also detected PEDV infection in these 324 intestinal samples. The results showed that 9.88% (32/324) of the intestinal samples were PEDV-positive (Table 3). Most (90.63%, 29/32) of the PEDV-positive samples were from clinically diarrheic piglets. Noticeably, 89.47% (17/19) of the PKV and PEDV double-positive pigs were clinically diseased. Meanwhile, 88.01% (257/292) of PEDV-negative pigs did not present enteric diseases, while 91.71% (177/193) of only PKV-positive but PEDV-negative pigs were also clinically healthy. In addition, only 11.98% (35/292) of PEDV-negative pigs were clinically diseased and only 8.29% (16/193) of PKV-positive but PEDV-negative pigs were from the group of diarrheic piglets. These results suggested that PKV infection in itself is less likely to cause enteric diseases.

### 3.3. PKV Strains Are Rapidly Evolving in Chinese Swine Herds

**Figure 3:**
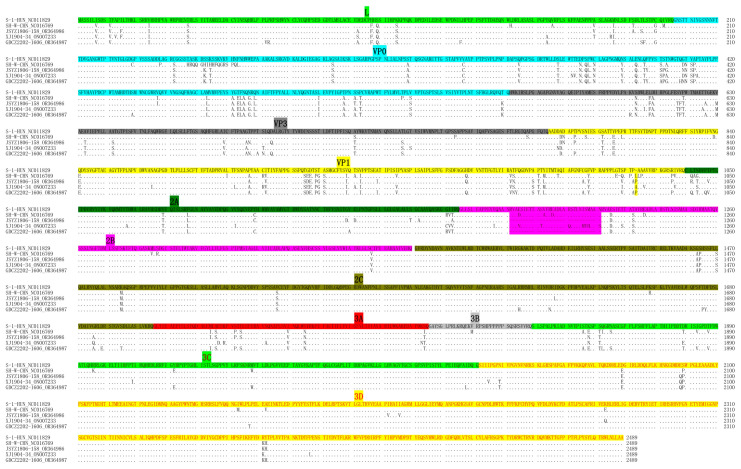
Multiple sequence alignment of PKV polyproteins. The aligned sequences include representative S-1-HUN and SH-W-CHN isolates and three PKV strains identified in this study. The aa insertion in VP1 is highlighted in yellow, while the 30 aa deletion in 3B is marked in pink. The proteins derived from the polyprotein are presented in distinct colors.

**Figure 4:**
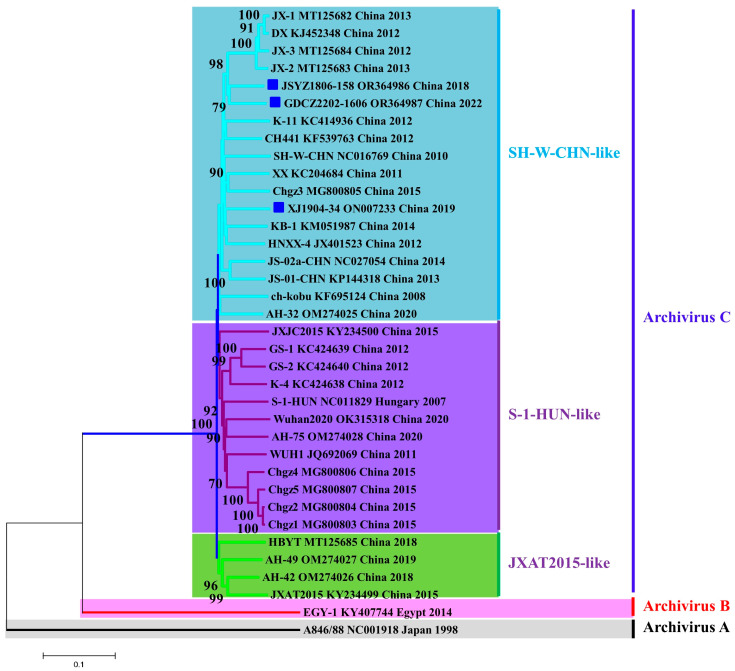
Phylogenetic tree based on 36 polyprotein-encoding sequences. The three species of kobuviruses are shown in distinct colors. Chinese PKV strains were clustered into three groups: SH-W-CHN-like, S-1-HUN-like and JXAT2015-like strains, respectively. All three PKV strains identified in this study are highlighted with blue squares. Each virus is shown by its name, GenBank accession number, country and year of isolation.

**Figure 5:**
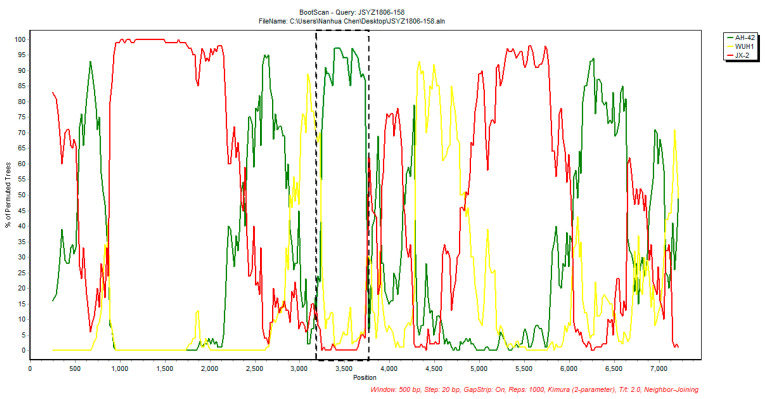
Recombination event detected in JSYZ1806-158 using SimPlot. The cross-over region is shown in the dashed line box, which is consistent with the result from RDP4. JX-2 is the major parental virus, while AH-42 is the minor parental virus for JSYZ1806-158. The y axis shows the percentage of permutated trees employing a sliding window of 500 bp and a step size of 20 bp. Other options, including Kimura (2-parameter) distance model, 2.0 Ts/Tv ratio, neighbor-joining tree model and 1000 Bootstrap replicates, are used.

To analyze the dynamic evolution of PKV in Chinese swine herds, three representative PKV-positive samples were submitted for genome sequencing, and the obtained nearly complete genomes were submitted to GenBank with the accession numbers OR364986, ON007233 and OR364987 (Table 4). Due to incomplete 5′UTR and 3′UTR sequences of available PKV genomes, the entire polyprotein-encoding sequences were used for multiple comparisons and phylogenetic analysis. Multiple sequence alignment showed that Chinese PKV strains contain an amino acid (aa) insertion at position 1023 of the polyprotein within the VP1 region compared with the S-1-HUN strain. In addition, the unique 30-aa-deletion in 3B was detected in the JSYZ1806-158 and GDCZ2202-1606 strains but not in the XJ1904-34 strain (Figure 3). Phylogenetic analysis showed that Chinese PKV isolates can be clustered into three groups: SH-W-CHN-like strains, S-1-HUN-like strains and JXAT2015-like strains, respectively. All three PKV genomes obtained in this study were grouped with SH-W-CHN-like strains, which are currently predominant in China (Figure 4). Polyprotein comparisons showed that our PKV strains share low nucleotide similarities (87.69~88.18%), but obviously higher aa identities (94.42~94.94%) when comparing with the representative SH-W-CHN isolate (Table 5). Selective pressure analysis showed that the polyproteins of Chinese PKV strains were predominantly under negative selection, while only one to five positive selection sites were detected via three methods (Table 6). Moreover, JSYZ1806-158 was detected as a recombinant virus from JX-2 and AH-42 strains using both RDP4 (Table 7) and SimPlot (Figure 5).

## 4. Discussion

Diarrheic diseases are huge threats to the global swine industry. Several enteric viruses including PEDV and TGEV have been identified as direct causative pathogens for diarrhea in piglets [31]. PKV is another commonly found enteric virus in both healthy and diseased piglets [1,14]. However, the role of PKV in clinical diarrheic diseases is still unclarified. A previous study speculated that PKV inoculation not only caused clinical signs including diarrhea, emaciation and nausea, but also resulted in pathological lesions including interstitial pneumonia, nephrosis and gastroenteritis in piglets [39]. The digestive tract of PKV-infected and diarrheic piglets showed obvious extravasated blood from the stomach, with mononuclear phagocytes and lymphocytes infiltrating the submucosa. However, there is a lack of good evidence supporting the idea that PKV causes gastrointestinal disease [40]. Even though PKV was commonly detected in non-diarrheic pigs, this is not evidence that PKV is not a gastrointestinal-disease-causing virus. It could indicate that PKV is not a sufficient cause of gastroenteritis in itself. Our previous study showed that PKV is the most abundant virus, followed by PEDV, in severely diarrheic neonatal piglets [32]. In addition, in vivo replications of PKV and PEDV are coincidental with the development of neonatal diarrheic diseases. Moreover, PKV and PEDV are both mainly distributed in intestinal samples [2]. All these results suggest that both PKV and PEDV are closely associated with diarrheic diseases in piglets. To further evaluate the prevalence of PKV in Chinese swine herds in recent years and explore the correlation of PKV and PEDV infection with clinical diseases, in this study, we determined the PKV and PEDV singular and coinfection statuses in intestinal samples collected from six provinces of China between 2018 and 2022. In addition, the evolution of Chinese PKV in diarrheic and healthy pigs was also explored.

The prevalence of PKV has been widely evaluated all around the world. PKV-positive rates have been found to range from 13.1% (33/251) to 99% (97/98) [14,21]. In China, the first PKV study showed that the PKV-positive rate is 30.12% (97/322) in fecal samples collected during 2006–2007 from <15-day-old piglets [13]. The epidemiological studies in Sichuan province showed that the positive rate of PKV is 57.02% (207/363), using stool samples collected from 2011 to 2015 [41,42]. A 2016 report showed that the PKV infection rate is 62.1% (126/203) in Gansu province [43]. A recent study based on 483 fecal samples collected in 2020 showed that PKV is the most prevalent virus, with a positive rate of 51.1% (247/483) [44]. In this study, we showed that the PKV-positive rate is 65.43% (212/324), using intestinal samples collected from six provinces between 2018 and 2022. Our up-to-date epidemiological results are consistent with previous studies showing that PKV is highly prevalent in different regions of China.

The coinfection of PKV with other enteric viruses is more common than singular PKV infection. The coinfection of distinct enteric viruses is frequently associated with severe clinical disease [23,45]. Previous studies have confirmed that PKV is commonly co-infected with PEDV, TGEV, porcine deltacoronavirus (PDCoV), porcine astroviruses (PAstVs), porcine rotavirus A (PRV-A), porcine sapovirus (PSaV) and porcine encephalomyocarditis virus (EMCV) [32,44,46,47]. In this study, we also showed that PKV is frequently co-infected with PEDV. Even though PKV could be detected in pigs with or without enteric diseases, our results provided a first clue that most of the PKV and PEDV double-positive pigs are clinically diarrheic, whilst most of the PKV-positive but PEDV-negative pigs are clinically healthy (Table 3). Taken together with our previous results that the dynamics of significant PKV and PEDV in vivo replications are concurrent with the development of diarrheic diseases in piglets [32], all these findings support that PKV is unlikely a direct gastroenteritis-causing virus but a potential opportunistic pathogen in enteric diseases.

To evaluate the evolution of PKV in Chinese swine herds, three representative PKV genomes were determined and submitted for phylogenetic analysis. Our phylogenetic tree based on all available Chinese PKV polyproteins first showed that Chinese PKV strains have evolved into three groups, while SH-W-CHN-like strains are predominant. Multiple sequence alignment showed that the 30 aa deletions in 2B could be identified in PKV strains from both diarrheic (JSYZ1806-158) and healthy (GDCZ2202-1606) pigs, suggesting that the unique deletion in 2B is unlikely a virulent determinant. Recombination events occurring in viral genomes could negatively impact viral phylogenetic reconstruction. A previous study indicated that more than half of the PKV genomes were detected as being recombinant, while the nucleotide position 2762-3759 was detected as a frequent recombination fragment [48]. Recombination analysis showed that JSYZ1806-158 is an inter-group recombinant from the SH-W-CHN-like JX-2 strain and the JXAT2015-like AH-42 strain. The potential cross-over region (3175-3517) of JSYZ1806-158 was also consistent with the previous study, which supported the hypothesis that recombination plays a critical role in the evolution of PKV in China [48]. All these results indicated that PKV is rapidly evolving in Chinese swine herds.

Even though the origin of PKV cannot be determined, a recent study deduced the origin of PKV using Bayesian stochastic variable selection (BSSVS) analysis [48]. They showed that Kobuvirus is an ancient virus that can be traced back to ~1500 years, while PKV is an emerging virus with a divergence time of ~1900 years. Their results suggested that PKV has been circulating and evolving for a long period of time. Noticeably, each fragment or entire polyprotein comparisons showed that our PKV strains share low nucleotide similarities with a Chinese representative strain but obviously higher aa identities, indicating that many mutations occurring in Chinese PKV polyproteins are nonsense mutations. Selective pressure analysis showed that Chinese PKV polyproteins are predominantly under purifying (negative) selection. These results supported that even though PKV is rapidly evolving in Chinese swine herds, the strong purifying selection might help maintain the antigenic stability of PKV. Considering the characteristics of PKV, including its long evolving history, stable antigenicity, and existence within both diarrheic and healthy pigs, we hypothesize that PKV seems to have found a balance between virus and host interaction for fitness of survival.

## 5. Conclusions

This study provides an up-to-date epidemiological clue that PKV is likely an opportunistic pathogen rather than a direct causative virus in enteric diseases. In addition, our results also supported that PKV is rapidly evolving in Chinese swine herds, but strong negative selection helps to maintain the antigenic stability of PKV.

## Figures and Tables

**Table 1:** Primers used for PKV genome amplification in this study.

Primer	Sequence (5′-3′)	Location ^1^	Amplicon (bp)
PKV-F1	TCGGCCCTCTCACCCTCTTTTC	24-45 ^2^	1368
PKV-R1	GCAGCAGCAGGTTCCCACCA	1372-1391	
PKV-F2	AATGGTTGGACTCCTACGGTGAACA	1222-1246	1608
PKV-R2	TCCAGTGAGTGGATTCATCACCCA	2806-2829	
PKV-F3	CGCGGTACTTACACCGTGTGGGA	2665-2687	1472
PKV-R3	TGGACAACTTCAGGAGGAGCTTCAA	4112-4136	
PKV-F4	TACTCTGCTACTAACAACTGCACCCACTTT	3943-3972	1426
PKV-R4	TGGAAGTGACCACAACTACCTGGGA	5344-5368	
PKV-F5	CCGCCCAGAACCTGTTGTGATCTA	5043-5066	1458
PKV-R5	GCACCACAGAGACCCTGGAAGGT	6478-6500	
PKV-F6	TTGACTGGGCGACCCTCCAA	6236-6255	1562
PKV-R6	TCGAATGTCATCAGGCACAAACCA	7774-7797	
PKV-F7	TTTGGCAACGAGACGTATGAGATGATT	7465-7491	744
PKV-R7	AATACAGAATAGAAAGTAAAGGACAGTCAGGGA	8176-8208	

^1^ The location was determined according to the S-1-HUN (NC011829) strain. ^2^ The primer was not designed from the beginning of genome due to a poly G structure at 5′UTR.

**Table 2:** PKV infection status in six provinces of China from 2018 to 2022.

Year/Location	Sample No.	PKV-Positive No. ^1^	PKV-Positive Percentages
Year			
2018	11	3	27.27%
2019	37	26	70.27%
2020	142	76	53.52%
2021	16	4	25.00%
2022	118	103	87.29%
Total	324	212	65.43%
Location			
Jiangsu	67	43	64.18%
Xinjiang	37	26	70.27%
Guangdong	32	19	59.38%
Henan	84	69	82.14%
Shandong	62	46	74.19%
Fujian	42	9	21.43%
Total	324	212	65.43%

^1^ PKV was detected via real-time PCR assay developed previously [32].

**Table 3:** The correlation of PKV and PEDV infection with enteric diseases.

Infection Status ^1^	Diarrheic Piglets <10 Days Old	Pigs without Diarrhea >1 Month Old	Total
PEDV+	29 (90.63%)	3 (9.37%)	32
PEDV+ PKV+	17 (89.47%)	2 (10.53%)	19
PEDV−	35 (11.98%)	257 (88.01%)	292
PEDV− PKV+	16 (8.29%)	177 (91.71%)	193

^1^ PKV and PEDV were detected via real-time RT-PCR assays [32].

**Table 4:** Background information for the representative PKV-positive samples used for genome sequencing.

No.	Name	Region (City, Province)	Date	Sample	Age	Symptom	PEDV
1	JSYZ1806-158	Yangzhou, Jiangsu	June 2018	Intestine	2 months old	Diarrhea	Negative
2	XJ1904-34	Kashi, Xinjiang	April 2019	Intestine	5 days old	Diarrhea, death	Positive
3	GDCZ2202-1606	Chaozhou, Guangdong	February 2022	Intestine	Adult	Clinically healthy	Negative

**Table 5:** Polyprotein-encoding gene and protein comparisons of our three PKV strains and reference SH-W-CHN isolate.

Region	Length (nt)	Nucleotide/Amino Acid Identities (%)					
		JSYZ1806-158 vs. SH-W-CHN	XJ1904-34 vs. SH-W-CHN	GDCZ2202-1606 vs. SH-W-CHN	JSYZ1806-158 vs. XJ1904-34	XJ1904-34 vs. GDCZ2202-1606	JSYZ1806-158 vs. GDCZ2202-1606
L	585	89.23/96.41	88.55/94.36	89.06/93.85	87.35/95.90	86.84/94.36	89.23/95.90
VP0	1098	87.81/92.90	86.99/92.90	86.72/92.08	87.80/97.81	87.80/97.54	88.07/98.63
VP3	669	85.05/93.72	84.16/93.27	84.01/92.38	86.40/96.86	86.55/95.96	91.03/98.65
VP1	765	82.61/90.98	86.14/91.76	82.09/90.59	83.01/92.16	84.58/92.55	88.76/98.82
Structural (VP0-VP3)	2532	85.51/92.54	85.99/92.65	84.60/91.71	85.98/95.85	86.49/95.62	89.06/98.70
2A	408	87.99/91.91	89.71/94.12	89.71/96.32	89.95/90.44	89.22/92.65	91.42/92.65
2B	495/585/495 ^1^	74.36/83.08	85.30/92.82	75.90/83.08	76.24/83.59	76.41/83.59	93.33/100
2C	1005	91.74/99.10	90.45/99.40	91.34/97.61	91.54/99.40	91.24/97.91	91.74/97.91
3A	270	90.37/98.89	86.30/90.00	92.59/98.89	88.52/91.11	87.78/91.11	93.33/100
3B	102	91.18/97.06	83.33/97.06	86.27/97.06	86.27/100	88.24/100	90.20/100
3C	576	85.94/94.79	85.07/94.27	90.28/98.44	89.06/98.96	86.46/94.27	87.67/94.79
3D	1407	93.46/98.72	93.11/98.29	94.39/98.72	92.89/98.50	93.46/98.93	94.39/99.15
Nonstructural (2A-3D)	4263/4353/4263 ^1^	88.74/95.52	89.41/96.34	89.92/96.07	89.13/95.59	88.86/94.97	92.28/97.82
Polyprotein	7380/7470/7380	87.69/94.58	88.18/94.94	88.05/94.42	87.93/95.70	87.90/95.14	90.93/97.97

^1^ Indicates the nucleotide length of JSYZ1806-158, XJ1904-34 and GDCZ2202-1606, respectively.

**Table 6:** Selection pressure analyses for Chinese PKVs.

Test Method	Gene	No. of Codons	dN/dS	Positive Selection	Negative Selection	Criteria
SLAC	Polyprotein ^1^	2489	0.182	1	1134	*p*-value threshold of 0.05
FEL	Polyprotein	2489		5	1641	*p*-value threshold of 0.05
FUBAR	Polyprotein	2489		1	1856	Posterior probability of 0.95

^1^ The selection pressure analysis is based on 33 polyprotein-encoding sequences from China.

**Table 7:** Potential recombination events in JSYZ1806-158 identified using RDP v.4.71.

Recombinant Virus	Parental Virus		Breakpoint ^1^			Score for the Seven Detection Methods Embedded in RDP4						
	Major	Minor	Region	Begin	End	RDP	GENECONV	BootScan	MaxChi	Chimaera	SiScan	3Seq
JSYZ1806-158	JX-2 (MT125683)	AH-42 (OM274026)	2A	3175	3517	2.714 × 10^−3^	- ^2^	1.696 × 10^−3^	8.341 × 10^−6^	9.6 × 10^−6^	1.324 × 10^−2^	-

^1^ The breakpoint position in the JSYZ1806-158 polyprotein sequence. ^2^ “-” indicates that the recombination event is not significant. The *p*-value cut-off was set at 0.01.

## Data Availability

Three obtained nearly complete PKV genomes have been submitted to GenBank with accession numbers OR364986, ON007233 and OR364987.
